# Aspects Regarding the Influence of Obesity on the Molecular Characteristics of Breast Tumors

**DOI:** 10.7759/cureus.26952

**Published:** 2022-07-17

**Authors:** Iuliana Pantelimon, Laurentia Nicoleta Gales, Rodica Maricela Anghel, Maria Iuliana Gruia, Irina Nita, Catalina Vali Matei, Delia Bodea, Andra Maria Stancu, Edvina Pirvu, Mihaela Corina Radu, Anca Irina Dumitrescu, Loredana Sabina Cornelia Manolescu

**Affiliations:** 1 Medical Oncology, Cantacuzino Hospital, Bucharest, ROU; 2 Oncology, Prof. Dr. Alexandru Trestioreanu Institute of Oncology, Bucharest, ROU; 3 Oncology, Medical Oncology Department, Elias University Emergency Hospital, Bucharest, ROU; 4 Public Health, Carol Davila University of Medicine and Pharmacy, Bucharest, ROU; 5 Oncology, Medical Oncology Department, Cantacuzino Hospital, Bucharest, ROU; 6 Oncology, Clinical Hospital Colţea, Bucharest, ROU; 7 Medical School, Carol Davila University of Medicine and Pharmacy, Bucharest, ROU; 8 Fundamental Sciences, Faculty of Pharmacy, Carol Davila University of Medicine and Pharmacy, Bucharest, ROU; 9 Microbiology, Parasitology and Virology, Faculty of Midwifery and Nursing, Carol Davila University of Medicine and Pharmacy, Bucharest, ROU

**Keywords:** her2, her 2, bmi, obesity, leptin, breast cancer pathology

## Abstract

The influence of excess adipose tissue on the evolution and prognosis of breast cancer has been evaluated in numerous papers over the years. The ways in which obesity can influence the development, progression, and prognosis of this neoplasia are complex and requires the design of new studies, both clinical and preclinical. The aim of this study is to highlight a possible correlation between obesity-specific tumor microenvironment markers (adipokine or leptin) and the different histological subtypes and aggressive characteristics of breast tumors. We prospectively monitored the prognostic values of 39 patients diagnosed with breast cancer who received oncologic-specific treatment or are in follow-up regarding some obesity markers.

Our analysis included parameters such as age, body mass index, immunohistochemical characteristics, and plasma concentration of leptin. The methodology was designed to reveal a possible correlation between obesity (quantified by measuring body mass index and waist circumference), the plasma level of leptin, and breast tumor immunohistochemical characteristics. The patients diagnosed with aggressive tumors subtypes (HER2-positive and triple-negative) had a significantly higher body mass index than patients diagnosed with luminal type tumors (32 kg/sqm versus 27 kg/sqm), the difference being 5 kg/sqm. In patients with non-luminal type breast tumors (HER2-positive and triple-negative), serum concentration of leptin is 55 pg/ml compared to 48 pg/ml in luminal type, statistically significant, p=0.0168. Leptin plays an important role in the connection of specific microenvironment tumors to breast cancer. An increased serum concentration of this adipokine was found in patients with HER2-positive and triple-negative breast tumors compared with luminal-type breast tumors, which could open new directions in the research of breast cancer prognosis in obese patients.

## Introduction

Breast cancer is a heterogeneous disease with known risk factors and a thorough molecular classification that includes hormonal receptor status and the assessment of Ki67 percentage for prognostics [1-3]. The genetic component has an important role and cannot be controlled, but other risk factors such as obesity, childbirth, or breastfeeding can. 

Obesity, body size, physical activity, calorie intake, smoking, alcohol, sexual life, and occupational exposures play an important role in determining a person’s risk of developing breast or cervical cancer [1-6]. 

Obesity is considered a chronic disease whose prevalence is constantly increasing in all age groups, the value of the body mass index increasing everywhere in the world [7]. The main tool for assessing obesity is body mass index (BMI) along with waist circumference (in people with a BMI between 24.9 and 30 kg/m²) [8].

Previous epidemiologic studies have consistently shown associations between obesity and increased risk for cancers of the endometrium, kidney, gallbladder (in women), breast (in postmenopausal women) [9], and colon (particularly in men) [7,8].

There are supporting data for the association between increased BMI and inflammatory breast cancer, regardless of menopausal status or the presence of estrogen receptors on immunohistochemistry [10]. Obesity expressed by BMI may be an independent prognostic factor in the evolution of breast neoplasms [11].

Adipose tissue consists mainly of adipocytes but also of fibroblasts, fibroblastic preadipocytes, endothelial cells, and immune cells and has endocrine and depot function, being essential in maintaining energy homeostasis. Adipokines connect obesity with breast neoplasm [12]. Leptin promotes angiogenesis, cell proliferation, migration, and invasion, and has a pro-inflammatory effect. It is an adipokine secreted by adipocytes, key cells in transformed breast epithelial cells [13].

There are three systems involved in the connection of adipose tissue and breast cancer: steroidal sex hormones, adipokines, and insulin-like growth factor 1, (IGF-1). Leptin induces the migration of vascular endothelial cells, by means of overexpression of metalloproteinases and the urokinase-type activator of plasminogen [14]. The impact of its increased serum level on breast tumor phenotypes and aggressiveness may open new directions for research of molecular mechanisms. Leptin works by stimulating neo-angiogenesis [15-19]. The presence of the leptin receptor and its overexpression to immunohistochemistry showed a higher rate of metastasis [20,21].

This study aims to identify a connection between excess adipose tissue assessed by calculating BMI and measuring waist circumference, serum level of adipokine leptin, and tumor immunohistochemical (IHC) characteristics. The demonstration of this connection can bring new information on the influence of excess adipose tissue and adipokines on the tumor microenvironment and on the interactions at the molecular level that could determine the immunohistochemical (IHC) profile of these tumors. We consider that due to the small number of patients included in this study, the serum value of leptin in non-obese patients with aggressive tumors is not considered statistically significant.

## Materials and methods

Data sources

This is a prospective, observational, non-randomized study of a sample of 39 patients, evaluated between January 2019 and October 2019, at the Elias University Emergency Hospital. The basis for the calculation of the sample size was the period of time, we included all women that came to our hospital for 10 months in 2019 and were within the criteria of inclusion. The sample obtained was a representative sample for the population of patients diagnosed with breast neoplasm in a center with experience in the diagnosis and treatment of oncological diseases.

A database was designed including the following data: patient age, main immunohistochemical (IHC) characteristics (percentage of estrogen and progesterone receptors, human epidermal growth factor receptor 2 (HER2), status and proliferation index Ki67, (antigen Ki67 is a nuclear protein that is associated with cellular proliferation), tumor stage (TNM), body mass index (BMI), waist circumference and serum value of leptin determined from patients' blood. The value of the body mass index was considered the one from the initial diagnosis, so, overweight and obese patients had associated with this comorbidity before cancer diagnosis.

Informed consent was obtained from the patients, and the study was approved by the ethical committee on human research of Elias Emergency Hospital Bucharest, Romania (Number 5748/13.08.2018).

Inclusion criteria

The inclusion criteria included patients diagnosed with stage I, II, or III invasive ductal/lobular carcinoma in the last two years, undergoing treatment or monitoring, absence of another type of associated neoplasia, receiving chemotherapy at a certain point in the therapeutic sequence, and those for whom all the data necessary to complete the database have been available

In the formed group, we paid special attention to infectious disease symptoms and we also screened for asymptomatic infections, like HIV or viral hepatitis before starting the systemic treatment (we did not include in our group any of these patients).

The protocol for the IHC technique included the following algorithm (we must mention that the tissue samples were done prior to our review):

Placing the section of the paraffin-embedded tissue from the sample's patients into a thermostat at a temperature of 57 °C for 60 minutes. Then they were transferred to alcohol baths, two in succession for three minutes each, to dissolve the paraffin. Then they were transferred onto glass slides suitable for immunohistochemistry. The slides were allowed to dry overnight and stored at room temperature until ready for use. The slides were left in this solution for 10 minutes at room temperature and then rehydrated by successive baths of de-paraffining agent (benzene, toluene, xylene, or other similar substances), with decreasing concentrations of 96%, and then successive baths of distilled water. Sections were kept in water until the antigen recovery process began. From this point on, we did not allow the sections to dry out because the binding of the antibodies wouldn't be specific. The number of baths and the exposure time varied depending on the manual or automatic process, with a minimum of 25 minutes.

For the next step, the antigens were recovered (unmasked) from the tissue sections by placing the sectioned slides in a vessel containing the solution for antigen recovery (Epitope Retrieval Solution, Dáko, Aptum Biologics Ltd., Southampton, UK) and kept at room temperature, a method that aimed to expose antigenic sites to allow antibodies to bind.

Then, we incubated in the microwave at 95-97 °C for 30 minutes. The blades were transferred to another vessel and washed thoroughly with tap water and then with distilled water.

The next step was to block the endogenous peroxidase with 3% hydrogen peroxide for five minutes to interfere with the detection of the signal. Without washing, but only by removing the blocking solution, incubation with monoclonal antibodies (antibodies to estradiol receptor (ER), progesterone receptor (PR), HER2/proto-oncogene Neu, Ki67) was performed. For cases with uncertain histological aspects, other antibodies were used to establish a definite diagnosis.

The protocol for the HER2/neu detection included the following algorithm:

HER2/neu is a monoclonal antibody that expresses the ERBB2 gene (HER2), being extremely useful for molecular classification of malignant breast tumors, prognosis assessment, and treatment administration. The positivity of this marker is reported in scores from 0 to 3+, being usual that for scores of 1+ and 2+ the testing is completed by in situ hybridization cytogenetics techniques. The method is based on the identification of a specific DNA sequence at the chromosomal level. Two chromogens are used, one for the labeling of centromeric chromosome 17 (CEP 17) and another for the labeling of the HER2 gene found at this level. According to the American Society of Clinical Oncology/College of American Pathologists (ASCO/CAP) recommendations for HER2 testing, the reaction is interpreted as positive for amplification at a HER2 ratio: CEP17> 2.

Statistical analysis

For statistical analysis, the program R, version 3.6.2 (2019) was used (R Foundation for Statistical Computing, Vienna, Austria). In addition to standard packages, the following packages were used from the R program were used: (1) Therneau T (2022). A Package for Survival Analysis in R. R package version 2.38; (2) Alboukadel Kassambara and Marcin Kosinski (2018) Survminer: Drawing Survival Curves using 'ggplot2'; R package version 0.4.3.

In this study, BMI and abdominal circumference were used as indicators for obesity and the analysis algorithm estimated possible correlations between them and tumor immunohistochemical characteristics. We used the correlation index Pearson's R / Spearman's p to make a comparative analysis of the studied group (obese vs. non-obese) with these indices, using (depending on the distributions of the variables) a bidirectional Welch t-test.

Plasma determination of leptin

Blood was withdrawn from the antecubital vein and immediately transferred to glass tubes containing Na2EDTA (1 g/L) and centrifuged at 4 °C. Plasma was kept frozen at -20 °C until analysis.

For the determination of plasma leptin, the Instant Human Leptin ELISA kit was used, which is an enzyme-linked immunosorbent assay, used for the quantitative detection of human leptin and was purchased from eBioscience manufacturers (ThermoFisher Scientific, Waltham, MA).

The principle of the test consists in fixing an anti-human antibody coated with leptin which is absorbed on the wells of the test plate. Human leptin present in the sample (serum, in our case) or standard binds to absorbed antibodies; another anti-human anti-leptin antibody conjugated with biotin binds to human leptin captured by the first antibody, streptavidin, immobilized with horseradish peroxidase (HRP) binds to biotin-conjugated anti-human leptin. This fixing process is performed in two stages. After incubation, anti-human leptin conjugated to unbound biotin and streptavidin-HRP is removed during a washing stage, and the HRP-reactive substrate solution is then added to the wells of the tested plate. A colored product is formed in proportion to the amount of soluble human leptin present in the sample. The reaction is finished by the addition of ELISA stop solution made with 100ul 1M H2SO4 and then the absorption is measured at a spectrophotometer at 450nm. In the end, we prepare a standard curve from seven standard dilutions of human leptin and determine the concentration of the human leptin sample.

## Results

The characteristics of the patients included in the study group are shown in Table 1.

**Table 1:** Characteristics of the patients. Stage T represents the tumor stage.

Characteristic		N (%)
Age at diagnosis	Under 50 years	13 (33.3%)
	50-70 years	22 (56.4%)
	Over 70 years	4 (10.2%)
BMI (body mass index)	18-25 kg/sqm	7 (20.51%)
	25-30 kg/sqm	14 (35.89%)
	Over 30 kg/sqm	18 (43.6%)
Waist circumference	Under 80 cm	4 (10.2%)
	81-88 cm	6 (15.4%)
	Over 88 cm	29 (74.4%)
Intrinsic subtype	Luminal A	8 (20.5%)
	Luminal B	19 (48.7%)
	Luminal B HER2 positive	5 (12.8%)
	HER2 positive	2 (5.2%)
	Triple negative	5 (12.8%)
Stage T	T1 (under 2 cm)	7 (18%)
	T2 (2-5 cm)	18 (46%)
	T3 (more than 5 cm)	1 (2.6%)
	T4 (invasion at the level of the chest wall or tegument)	13 (33.4%)
Invasion of regional nodes	Present	30 (77%)
	Absent	9 (23%)

Although in this group the patients were not selected according to the body mass index, a significant percentage of overweight and obese patients was found. Thus, we observed that the percentage of patients with obesity is 43.6%, and 35.89% for those who are overweight. In conclusion, 79.49% of the patients in this group come with an excess of adipose tissue. We considered height and weight established at initial diagnosis. The waist circumference at the time of signing consent was more than 88 cm in most patients (74.4%). Around 77% of these patients presented invasion in the axillary nodes, this indicates an advanced stage at diagnosis. Although it is considered that obese patients more frequently have locally advanced tumors due to the increased size of the mammary gland, in this study the report was in favor of patients with staged T1-T2 tumors (61%), regardless of body mass index.

Influence of excess adipose tissue on breast cancer characteristics

Estrogen Receptor Status and Obesity

Table 2 presents the data resulting from the statistical calculation of Pearson’s R correlation index to establish a possible influence of excess adipose tissue on the expression of estrogen receptors expressed on tumor cells.

**Table 2:** Pearson's R correlation index between the presence of estrogen receptors in excess adipose tissue Assessed by measuring BMI and waist circumference.

Obesity Parameter	Estrogen Receptors Pearson's R (p-value)
BMI (kg/sqm)	-0.01 (0.9288)
Abdominal circumference (cm)	-0.07 (0.6551)

No statistically significant correlations were identified between the analyzed data (p > 0.05). The descriptive analysis of the level of estrogen receptor expression in the groups showed that they are not significantly altered (Table 3).

**Table 3:** Evaluation of the statistical significance of estrogen receptor expression when comparing obese versus non-obese patients. SD: standard deviation; IQR: interquartile range

Estrogen Expression (%)	Median Group with Obesity (BMI-kg/sqm)	Median Group Without Obesity (BMI-kg/sqm)
Mean ± (SD)	66.83 ± 38.10	71.00 ± 36.51
Median (IQR)	87.50 (30.00)	90.00 (25.00)
Min to Max	0.00 to 100.00	0.00 to 10.00
Skewness	-1.11	-1.35

We observed that distributions of the level of estrogen receptor expression in both groups are far from the normal distribution, the median in the group with obesity was 87.5 and in the group without obesity was 90, having a bimodal character. To see if the difference is of statistical significance, a Wilcoxon rank-sum test was used, the difference was 2.50 with a confidence interval (CI 95%: 10.00 to 5.00). The data obtained from the calculations demonstrate that the differences are not statistically significant (p > 0.05). In this regard, comparative boxplots can be tracked.

**Figure 1:**
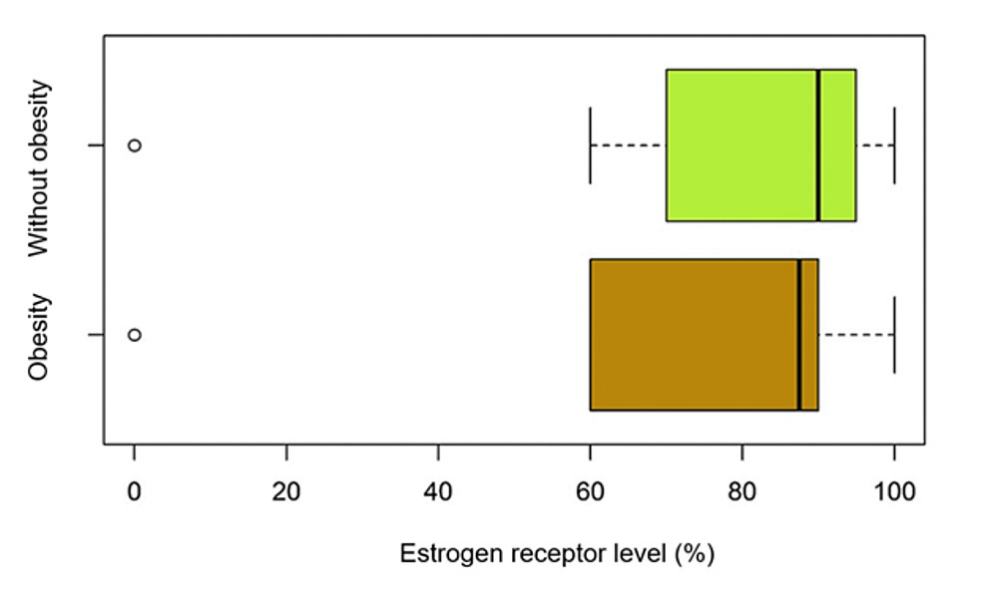
Comparison of estrogen receptor expression within the group of patients with obesity versus the group without obesity.

The graphs and tables above show that the expression level of estrogen receptors in the two groups of patients with/without obesity are not different and therefore does not appear to be influenced by excess adipose tissue (Figure 1).

Obesity and proliferation index Ki67

Since the role of the Ki67 proliferation index as an aggressiveness factor in the evolution of breast neoplasm is supported by several studies, we analyzed how excess adipose tissue influences its value [3].

The analysis of the influence of obesity on Ki67 expression level (correlation indices r Pearson BMI/abdominal circumference vs Ki67 expression level) is presented in Table 4.

**Table 4:** Evaluation of the proliferation index in obese patients (BMI/abdominal circumference).

Obesity Parameter	Expression Ki67 Pearson's R (p-value)
BMI (kg/sqm)	0.080 (0.6255)
Abdominal circumference (cm)	0.071 (0.6655)

No correlations with statistical significance were highlighted (p> 0.05). Virtually no correlation was demonstrated between the Ki67 proliferation index value and the two obesity parameters (Ki67 proliferation index and waist circumference) used in this study. Probably, in the evolution of breast neoplasm in patients with excess adipose tissue, molecular mechanisms involved do not influence this proliferation index [22].

To investigate whether there are statistically significant differences in the proliferation of index Ki67 between the two groups, a Wilcoxon Rank Sum test was used; the median in the group with obesity was 30 and in the group without obesity was 25, p-value = 0.9661, Difference (CI 95%)=5.00 (-15.00 to 10.00). The observed differences are not statistically significant (p> 0.05).

Distribution of HER2-positive breast tumors according to the presence/absence of obesity

Since HER2 overexpression is both a negative prognostic factor and a predictive factor of the response to anti-HER treatments, we further introduced the comparative analysis between the two groups of patients (with/without obesity), depending on the positive HER2 cases. Comparative analysis between groups, according to HER2-positive cases is presented in Table 5.

**Table 5:** The proportion of HER2-positive breast tumors in obese versus non-obese patients.

HER2	Group with Obesity	Group without Obesity
Positive (%)	4 (22.22)	3 (14.29)
Negative (%)	14 (77.78)	18 (85.71)

The comparative analysis of HER2 positive and negative breast tumors within the two groups of obese and non-obese patients shows a higher preponderance of the histological subtype HER2 positive for immunohistochemistry in obese patients, p-value=0.6825, Difference (CI95%)=0.08 (-0.16 to 0.32). There are no statistically significant differences between obese and non-obese patients (p > 0.05).

Comparative analysis of the prevalence of triple-negative breast tumors in the two groups of patients (obese/non-obese)

A real problem in the therapeutic management of breast cancer is the triple-negative subtype. Comparative analysis between groups, depending on triple-negative cases is presented in Table 6.

**Table 6:** The distribution of the percentage of triple-negative tumors in the two patient groups (with/without obesity).

Triple Negative	Group with Obesity	Group without Obesity
Yes - No. (%)	3 (16.67)	2 (9.52)
No - No. (%)	15 (83.33)	19 (90.48)

Studying the data represented in the graph above, it is highlighted that, in the group of patients with obesity, a greater number of them were diagnosed with triple-negative breast tumors.

To see if the differences are statistically significant, an exact Fisher test for the proportion of triple-negative patients in the two groups was used, p-value = 0.6466, the difference (CI 95%) = 0.07 (-0.14 to 0.28); the proportion of triple-negative group with obesity was 0.17 and the proportion triple-negative group without obesity was 0.10. The differences are not statistically significant (p > 0.05) as the results are in the context of a small group of patients.

Since leptin plays an essential role in the molecular mechanisms involved in the evolution of breast neoplasms in patients with obesity, we tried to establish correlations between its serum level, the presence of excess adipose tissue, its association with certain histological subtypes, and its influence on the prognosis of patients in the study group.

Analysis of the influence of obesity on serum levels of leptin (correlation indices Pearson's R BMI/abdominal circumference versus leptin).

**Figure 2:**
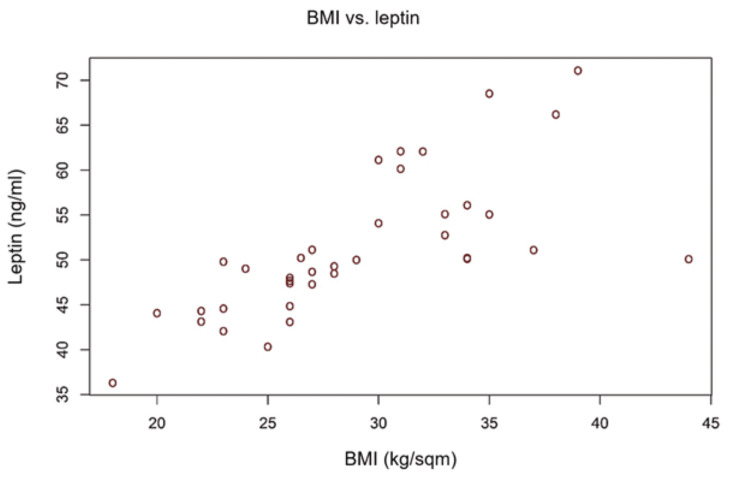
Correlation between BMI and serum levels of leptin

**Figure 3:**
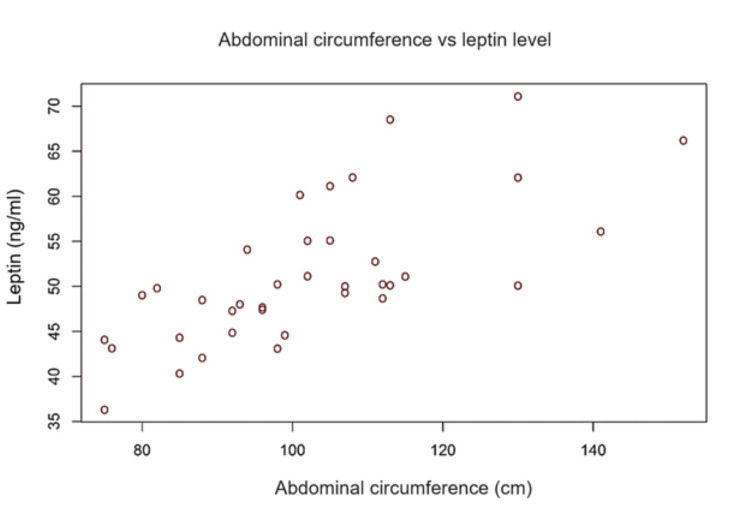
Correlation between the abdominal circumference and serum level of leptin.

There were two strong positive correlations with statistical significance between leptin level and body mass index/Abdominal Circumference. In our study, the serum leptin correlates with the presence of high adipose tissue evaluated by calculating the body mass index or measuring the abdominal circumference. This issue is demonstrated by the calculation of the Pearson correlation index (Table 7), which has a statistically significant value. The graphs are suggestive in illustrating the obesity-leptin connection (Figures 2-3).

**Table 7:** Calculation of Pearson correlation index between obesity (BMI/waist circumference) and serum leptin level.

Obesity Parameter	Leptin Pearson's R (p-value)
BMI (kg/sqm)	0.710 (< 0.0001)
Abdominal Circumference (cm)	0.702 (< 0.0001)

**Figure 4:**
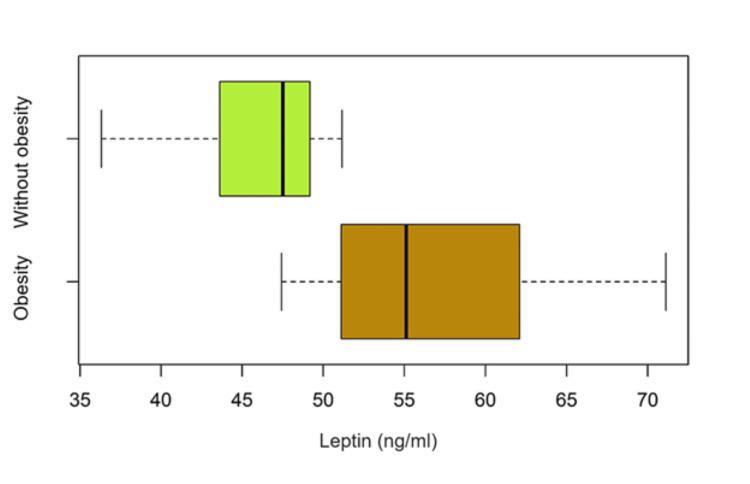
Comparison of serum levels of leptin in the two groups of patients (obese and non-obese)

In Table 8 and boxplots (Figure 4) the mean value and the median value of serum leptin are compared between obese and non-obese patients. We also applied the Welch test that confirmed a statistically significant difference (p < 0.05).

**Table 8:** Statistical significance of serum level of leptin for the two groups (Welch test). CI – confidence interval.

Mean value of serum leptin (ng/ml) in Group with Obesity	Mean value of serum leptin (ng/ml) in Group without Obesity	P-value	Difference [CI95%]
57.28	46.13	< 0.0001	11.15 [7.18 to 15.10]

To assess the association of obesity and elevated serum leptin values with tumor subtypes with an unfavorable prognosis, the groups of patients surveyed in this study were divided into two groups: group A in which patients with luminal tumors were included (luminal A and luminal B) and group B (HER2 positive and triple-negative tumors).

Comparative analysis of BMI within the Patient A and B groups (luminal/non-luminal)

The median value of the body mass index for group A (luminal) is 27 kg/sqm compared to patients in group B (non-luminal) whose median value was 32 kg/sqm (Table 9).

**Table 9:** Median body mass index values by luminal (group A) or non-luminal (group B) subtype. CI – confidence interval.

Median BMI (kg/sqm) Group A	Median BMI (kg/sqm) Group B	P-value	Difference [CI95%]
27.00	32.00	0.0762	-5.00 [-8.00 to 0.50]

**Figure 5:**
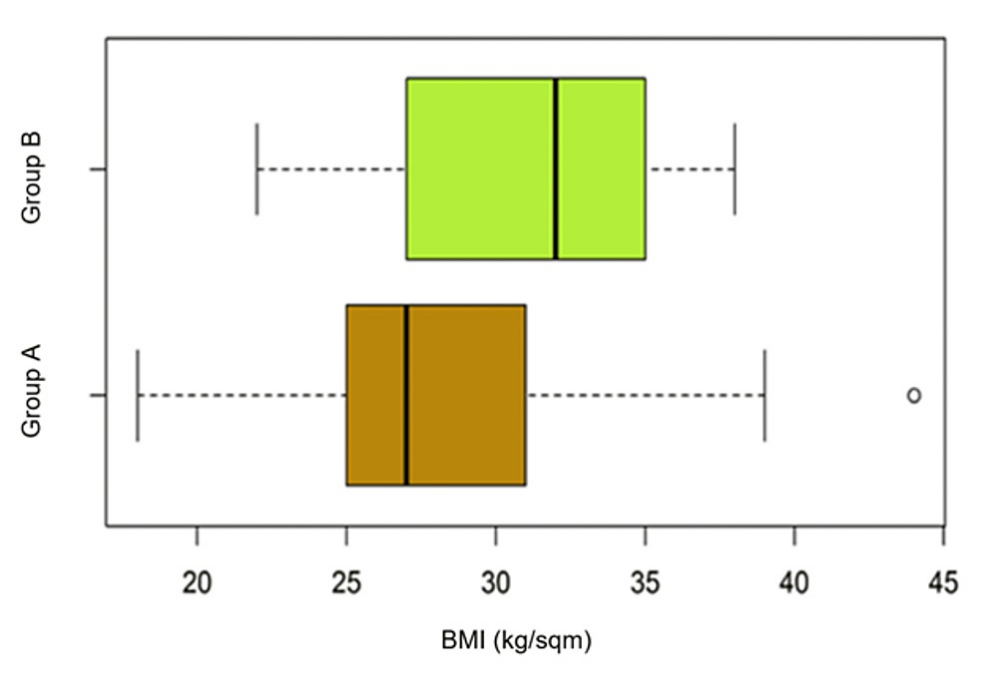
Body mass index in patients with luminal breast tumors (group A) compared to non-luminal tumors (group B)

We thus observe a difference of 5 kg/sqm between the two groups, which from a statistical viewpoint has no significance (marginal p-value, insignificant at 0.07). While the boxplots highlight an important difference in median body mass index values between the two groups, it is not statistically significant (Figure 5).

Comparative assessment of the median serum value of leptin between the two groups

In order to analyze a possible association between elevated serum leptin levels and intrinsic histological subtypes with an unfavorable prognosis, an analysis of the median leptin concentration in serum collected from patients included in group A (luminal) was performed compared to group B (non-luminal). Following the division of the group being studied into two groups according to the intrinsic subtype, respectively group A (luminal) and group B (triple-negative and HER2 positive), as well as the analysis of the median value of serum leptin a statistically significant difference is found (p < 0.05) between the two groups (Table 10). See also the comparative boxplots.

**Table 10:** Serum leptin median values by luminal (group A) or non-luminal (group B) subtype. CI – confidence interval.

Median value serum leptin (ng/ml) in Group A	Median value serum leptin (ng/ml) in Group B	P-value	Difference [CI95%]
48.86	55.09	0.0168	-6.23 [-12.45 to -0.95]

**Figure 6:**
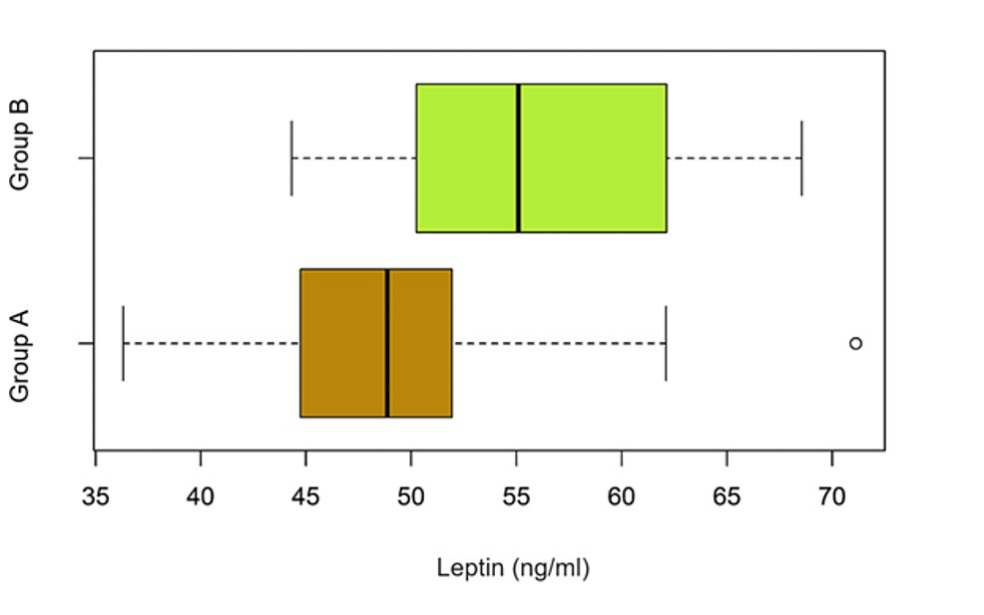
Serum leptin concentration in patients with luminal breast tumors (group A) compared to non-luminal tumors (group B).

In Figure 6 we illustrate that intrinsically aggressive subtypes of localized breast cancer, such as triple-negative or HER2 positive, have significantly higher levels of serum leptin compared to less aggressive subtypes such as luminal. The median serum concentration of leptin was 48.86 ng/ml for the luminal breast cancer subtypes compared with 55.08 ng/ml for the non-luminal subtypes.

## Discussion

This study is a scientific approach to the interaction between the tumor microenvironment rich in adipocytes and the influence of leptin on the development of breast tumors with an unfavorable prognosis (HER2 positive and triple negative).

The evaluation of the obesity status was done by measuring BMI index and waist circumference as data from literature found a significant association between both premenopausal and postmenopausal cancer risk and central obesity [23-24].

Molecular mechanisms are activated by leptin, as adipokine with a central role in the connection between breast neoplasm and obesity has been discovered [25]. There is an absolute 5% increase in mortality for obese patients with estrogen receptor-positive tumors [26]. One of the key questions that many breast cancer researchers investigated was whether obesity represents a risk factor only for some breast cancer subtypes [27].

No difference in estrogen receptor expression between obese and normal-weight patients could be demonstrated in our study. Our results suggest that receptors expressed on the surface of tumor cells do not change according to the metabolic slippages in patients with obesity. There are numerous studies in the literature that support the hypothesis that excess adipose tissue influences both the development of breast tumors that have estrogen receptors and those that do not have the same [28].

The Ki67 cancer proliferation index is a validated prognostic factor in the evolution of breast cancer, being one of the surrogate elements in the establishment of the intrinsic type (luminal A, luminal B, HER2-positive, triple negative). Ki67 proliferation index has a predictive value of overall survival and disease-free survival [29]. In our study, we could not demonstrate a difference in obese/non-obese patients. The Ki67 or MIB1 proliferation index is quantified by assessing the percentage of cells in the G1 phase of the cell cycle. The increased value shows a highly proliferating tumor, being an index of its aggressiveness. The fact that the Ki67 proliferation index is not statistically significant in obese patients may be due to the number of patients included in the study. It is not neglected that a higher number of HER2 positive and triple negative tumors were identified in the group of obese patients, these histological subtypes being known to have an increased Ki67 proliferation rate. (The median value of BMI index for group A (luminal) is 27 kg/sqm compared to patients in group B (non-luminal) whose median value was 32 kg/ sqm.)

When evaluating the presence of breast tumors that overexpress HER2 and triple negatives to verify whether these histological subtypes are present or not, a higher number of triple-negative and HER2-positive tumors was found in patients with obesity, but there was no statistically significant difference. A study that followed 329 patients with metastatic breast cancer found no impact of obesity on the clinical outcome of these patients [30].

There are numerous studies supporting the role of leptin and its receptor in the development and progression of breast cancer [31]. The first step to clarify the situation in our study group was to establish a correlation between the indicators of obesity and the serum level of leptin. A statistically significant correlation (p <0.0001) was observed. The plasma concentration of leptin is directly proportional to the volume of adipose tissue. Through its interaction with its stimulus receptor, this adipokine can induce increased cell proliferation, motility, and neo-angiogenesis [19]. The second step was dividing the patient group into two groups: group A (luminal subtype tumors with estrogenic receptors present and favorable prognosis) and group B (HER2-positive or triple-negative aggressive subtypes). It was thus determined whether obese patients are predominantly diagnosed with aggressive non-luminal tumors (HER2 positive or triple negative). In group B (non-luminal) there was an increase in the value of BMI index by 5 kg/sqm compared to group A (luminal), an argument in support of the hypothesis that obesity and excess adipose tissue are associated with aggressive tumor phenotypes. The third step demonstrated an association between elevated leptin levels in group B (non-luminal) versus group A (luminal). A median serum concentration of 55.09 ng/ml was found in group B compared to 46.86 ng/ml in group A (luminal). It appears that leptin and its receptor also play an important role in maintaining the proliferative and renewal capacity characteristic of stem cells in triple-negative breast tumors, according to a study using a murine model [32].

Serum leptin, the main adipokine involved in the connection between obesity and breast neoplasm was identified as a prognostic factor and statistically significantly increased values were identified in the serum of patients with non-luminal tumors, these subtypes having a poor prognosis. There are new data from interventional studies that explain how weight loss intervention and physical activity influence the prognosis of breast cancer patients [33]. One of the most important studies showed that the treatment of obesity can reduce the risk of breast cancer (four-year study, 693 overweight or obese patients with breast cancer). So a behavior weight loss intervention can lead to clinically meaningful weight loss in overweight/obese survivors of breast cancer [34].

Limitation of the study

This study was subject to several potential limitations. Firstly, the study was limited by the complexity of the way in which obesity can influence the development, progression, and prognosis of breast tumors. Consequently, there is a limit to the representativeness of the study sample, coming from the selection of respondents. This category of patients that display all the studied features is particularly scarce, which could explain the limited number of participants. Our institution is a large hospital in a metropolitan city where patients are predominantly from urban areas. This may be a weakness of the study because we are often faced with patients from urban areas, known to be more frequently obese than those from rural areas. Another limitation of this study is the fact that multiple determination of serum leptin was not performed (before/after therapeutic interventions). Obese patients have usually many comorbid-associated conditions and a high risk of viral infectious diseases; therefore we did not include them in our cohort; additionally, we did not include patients with asymptomatic infections before starting the systemic treatment [35-37].

## Conclusions

Obesity and excess adipose tissue are mainly associated with aggressive breast cancer subtypes, respectively HER2-positive and triple-negative. Leptin plays an important role in the connection to the tumor microenvironment specific to breast cancer. An increased serum concentration of this adipokine was found in patients with HER2-positive and triple-negative breast tumors compared with luminal-type breast tumors, which could open new directions in the research of breast cancer prognosis in obese patients. There are probably also other cellular pathways involved in the interaction between obesity and breast cancer carcinogenesis that do not implicate the estrogen receptors.
